# Robust Evolutionary-Game-Based Routing for Wireless Multimedia Sensor Networks

**DOI:** 10.3390/s19163544

**Published:** 2019-08-14

**Authors:** Md Arafat Habib, Sangman Moh

**Affiliations:** Department of Computer Engineering, Chosun University, 309 Pilmun-daero, Dong-gu, Gwangju 61482, Korea

**Keywords:** wireless multimedia sensor networks, routing protocol, evolutionary game theory, energy efficiency, end-to-end delay, packet delivery ratio

## Abstract

Nowadays, wireless multimedia sensor networks (WMSNs) are used in various applications. An energy-efficient and robust routing protocol is essential for WMSNs because the quality of service is important for traffic-intensive multimedia data, such as images and videos. A WMSN with multiple sinks allows cluster heads (CHs) to deliver the collected data to the nearest sink, thereby mitigating the delivery overhead. In this study, we propose a novel evolutionary-game-based routing (EGR) protocol for WMSNs with multiple sinks, in which the evolutionary game theory is exploited for selecting CHs. In EGR, an algorithm to mitigate data redundancy, based on the overlapping field of views of the multimedia sensor nodes, is also presented. This algorithm decreases the number of redundant transmissions, thereby increasing energy efficiency and network performance. According to the performance evaluation results of this study, the proposed EGR significantly outperforms the state-of-art protocols in terms of energy efficiency, end-to-end delay, packet delivery ratio, cluster formation time, and network lifetime.

## 1. Introduction

A wireless multimedia sensor network (WMSN) consists of wirelessly interconnected devices capable of retrieving multimedia contents, such as videos, audios, still images, and scalar sensor data from the environment [1,2,3]. The improvement and miniaturization of hardware can facilitate the development of sensor devices equipped with audio-visual multimedia modules [4]. Recently, the availability of cheap hardware, such as cameras and microphones, has had a great impact on the development of WMSNs. According to [5], a WMSN is a network of wirelessly interconnected sensor nodes that have multimedia devices and can retrieve video and audio streams along with the scalar sensor data. WMSNs can facilitate a wide range of applications in both public and military sectors. Surveillance sensor networks, law-enforcement reports, traffic control systems, advanced health care delivery, automated assistance to elderly telemedicine, and industrial process control are examples of such applications. In these applications, multimedia sensor nodes increase the amount of collected information, enlarge the range of coverage, and enable multi-resolution views [5]. Furthermore, multimedia sensor nodes collect snapshots and streaming multimedia contents. Snapshots are captured because of any event-triggered observation over a short period of time [4]. In contrast, streaming multimedia contents are captured and generated because of any event-triggered observation over a longer period of time. It is essential for WMSNs to have a strong foundation of hardware to satisfy the quality-of-service (QoS) requirements and fulfill application-specific demands.

WMSNs require higher bandwidth as compared to traditional wireless sensor networks (WSNs). For example, Crossbow [6], which is an IEEE 802.15.4 compliant WSN platform, has a data transmission rate of 250 kB/s, which is very low for high-end multimedia sensors. WMSNs are required to be energy-efficient to support high data rate; thus, more careful considerations should be given to conserve energy. Multimedia sensors produce large amount of data that require extensive processing, which consumes more energy and affects the network lifetime. Moreover, multimedia sensor nodes have a different concept of sensing region as compared to traditional WSNs. They have field of views (FoVs) and can capture images within a particular region. These operations are different from the sensing operations in traditional WSNs. The term FoV refers to the directional view of a multimedia sensor. The target object covered by a camera can be at a distant position. The images captured by the camera depend on the relative position and orientation of the camera toward the targeted object [7,8,9].

Over the last few years, many routing protocols have been proposed for WMSNs, which focus on energy efficiency, delay, and reliability. The real-time power aware routing (RPAR) protocol [10] dynamically adjusts transmission power and routing decision according to the network load and data packet size. Its unique forwarding and neighbor management mechanisms effectively save energy while meeting real-time constraints. However, RPAR fails to consider the energy hole and congestion problems. The routing protocol discussed in [11] adopts a cross-layer design approach between the network and MAC layers to distinguish between communication flows with different delay and reliability demands. However, it cannot control redundant data, which decreases energy efficiency and increases communication congestion. The distributed aggregate routing algorithm [12] determines multiple paths for multiple sinks while ensuring reliability. The operator calculus-based routing protocol [13] uses operator calculus methods on graphs to overcome the multi-QoS constrained routing problem. In this protocol, a node chooses the set of eligible next hops based on the given constraints and the distance to the sink. However, it ignores the impact of estimation accuracy on delay and reliability. The use of multiple sinks can be advantageous for WMSNs. It is necessary to deploy multiple sinks in the network to make it robust to sink failure. That is, a node has length-bound paths to at least two sinks. If any multimedia sensor node fails to send data because of sink failure, it can send data to another sink node alternatively. Furthermore, multiple sinks are helpful in implementing advanced applications and programming abstractions for routing algorithms and avoiding network congestions. So far, two routing protocols have been proposed for WMSNs that implement multiple sinks. In [14], multi-sink aware operations were integrated into an opportunistic routing framework to reduce energy consumption. Tong et al. [15] proposed CodeMesh, a coding-aware cross-path anycast routing protocol, to maximize the lifetime of time-driven multi-sink networks. CodeMesh combines proactive and reactive protocol features while benefiting from multiple sinks. Its route establishment does not rely on clock synchronization. However, it lacks a scheduling mechanism to improve real-time performance.

Recently, game theory has become a popular and useful tool in WSNs. This is because game theory can be widely used for modeling the interactions among entities with conflicting interests, which are competent to each other in a resource-constrained scenario. Four types of games have been used for routing in WSNs, which are cooperative games, non-cooperative games, evolutionary games (EGs), and reputation-based games [16]. In EG, the behavior of numerous agents that repeatedly engage in strategic interactions is studied. In EG, there are many behaviors involving the interaction of multiple organisms in a population. The success of any organism is highly dependent on how it interacts with others [16]. Therefore, the fitness of an organism should be measured in the paradigm of the full population and not individually. Two of the biggest advantages of using EG to design routing protocols for WMSNs are that cluster heads (CHs) can be elected optimally and it can exploit energy imbalance among the nodes in EG formulation. Many routing protocols have been designed for traditional WSNs using evolutionary game theory. Two of the prominent protocols are game-theory-based energy efficient clustering routing protocol (GEEC) [17] and energy-efficient routing protocol based on evolutionary game (EEREG) [18]. However, for WMSNs, there is only one routing protocol that is partially based on game theory. A combination of game theory and ant colony algorithm is presented in [19] to overcome the problem of QoS routing in WMSNs. This combination is adopted to overcome the drawback of the current ant-based routing protocols for WMSNs. In this routing algorithm, game theory is based on the assumption that the sensor nodes are rational and opt for selfish actions (maximizing pay-offs at minimum costs). However, the study does not provide any performance evaluation of the proposed routing scheme. To the best of our knowledge, no routing protocol using EG for WMSNs has been reported till now in the literature.

In this study, an EG-based routing (EGR) protocol for WMSNs with multiple sinks is proposed, which is a cluster-based routing protocol with the intelligent mechanism of CH election. Clusters are formed based on the overlapping FoVs of the nearby sensor nodes. For every cluster, a CH is elected using EG. Furthermore, a data redundancy avoidance mechanism is also proposed. The overlapping area of the FoV of an object shared by two nearby sensor nodes is calculated, and it is decided if the captured data is redundant. This approach decreases the number of redundant transmissions, thereby increasing energy efficiency and network performance. The proposed EGR is compared with the five protocols. They areGEEC, EEREG, QoS-aware multi-sink opportunistic routing (QMOR) [14], a routing protocol designed by Alaei and Barcelo-Ordinas [20] that we will refer to as AB routing in this study, and low-energy adaptive clustering hierarchy-centralized (LEACH-C) [21]. According to the performance evaluation results of this study, the proposed EGR outperforms GEEC, EEREG, QMOR, AB routing, and LEACH-C in terms of energy efficiency, end-to-end delay, packet delivery ratio, cluster formation time, and network lifetime. The proposed EGR exhibits 31.7%, 32.9%, 66.8%, 42.2%, and over 200% less end-to-end delay as compared to GEEC, EEREG, QMOR, AB routing, and LEACH-C, respectively.

The contribution of this study can be summarized as follows:

- The unique and intelligent method of CH election using EG makes the proposed EGR energy-efficient. A node with the highest level of residual energy in each cluster becomes the CH in each round. EG has been used in cluster-based routing protocols for WSNs in the literature; however, the proposed EGR is the first attempt to use EG for routing in WMSNs.
- A technique for avoiding data redundancy based on the calculation of the region overlapped by two nearly placed sensors is introduced, which results in a significant increase in the network lifetime.
- The proposed EGR makes the delivery of the sensed data faster and more reliable by effectively exploiting the multi-sink environment.
- The proposed EGR outperforms the conventional routing protocols in terms of energy efficiency, end-to-end delay, packet delivery ratio, and network lifetime.

A lot of symbols have been used in this paper to present different concepts and mathematical formulas. To increase the readability of the paper, we summarize all the symbols in Table 1.

The remainder of this paper is organized as follows: In the following section, the related works are summarized and discussed in brief. In Section 3, the network model and problem statement imposed in this study are presented. In Section 4, the proposed EGR is presented in detail. That is, the cluster formation process, CH election process using EG, data redundancy avoidance algorithm, and routing algorithm are presented. In Section 5, the performance of the proposed EGR is evaluated via extensive simulation and compared with QMOR, AB routing, and LEACH-C protocols. Finally, this study is concluded in Section 6.

## 2. Related Works

In this section, we review the routing protocols designed for WMSNs. Several routing protocols have been proposed that exploit different types of methods to achieve data reliability and energy efficiency to facilitate WMSNs.

In [22], an ant-based service-aware routing algorithm (ASAR) for WMSNs was proposed. The routing algorithm opts for the appropriate paths for variant QoS requirements. The routing scheme is mainly based on the data transfer between CHs and sink node. The CHs transfer different classes of data. To quicken the convergence of the algorithm and optimized network resources, the proposed algorithm (also discussed in [22]) quantifies the phenomenon value on the sink node to reduce transmission frequency and control messages.

A combination of game theory and ant colony algorithm was presented in [19] to overcome the problem of QoS routing in WMSNs. This combination is adopted to overcome the drawback of the current ant-based routing protocols for WMSNs, which is the long time required by the forwarding ants to determine the destination and overhead occurred because of the use of many backward ants that update the routing probability distribution. In this routing algorithm, game theory is used under the assumption that the sensor nodes are rational and opt for selfish actions (maximizing pay-offs at minimum costs). However, the study does not provide any performance analysis.

An image transmission framework for the optimization of the perceptual quality and energy expenditure in WMSNs was proposed in [23]. The goal of the proposed scheme was to ensure the perceptual quality at the end user using an analytical distortion model. The model could predict the image distortion resulting from any error pattern. The outstanding part in [23] is the use of a content-aware packet prioritization along with an energy and delay aware routing protocol.

A QoS-aware routing protocol was presented in [24] that supports high data rate for WMSNs and ensures the bandwidth, end-to-end delay requirements of real-time data. The routing scheme proposed in [24] uses multiple paths, channels, and QoS packet scheduling techniques based on dynamic bandwidth adjustment and path-length-based proportional delay differentiation techniques to fulfill the bandwidth and delay requirements, respectively. The bandwidth and delay requirements are adjusted locally at each node. The nodes discussed in [24] are homogeneous and are responsible for performing application-specific tasks, such as video, audio or scalar data acquisition. A protocol proposed in [24] improved the average delay per real-time packet, average lifetime of a node, and throughput of non-real-time data.

A dynamic node collaboration scheme based on FoV for a target tracking application was presented in [25]. Unlike traditional sensing models, it is a nonlinear localization-oriented sensing model for WMSNs. It is based on clustering and exploits Monte Carlo techniques to determine the target locations cooperatively. This protocol was intended for mobile target tracking using camera sensors by considering perspective projection and observation noises.

In [26], a mathematical model for QoS-aware route determination method for WMSNs is reported. The mathematical model is used to find optimal path for providing appropriate shared radio that satisfies the QoS for a wide range of real-time intensive media. To manage hop-by-hop switching in the proposed routing scheme, a mathematical model based on the Lagrangian relaxation method is used. Objective functions with embedded criteria are used to decide the pathway from the source to the sink.

A new routing mechanism for WMSNs based on software defined networking (SDN) technology is introduced in [27]. SDN can facilitate with the visibility of network resources and programmable interfaces. The routing protocol devised in [27] is basically a QoS-aware routing mechanism for WMSNs. The protocol could achieve high throughput for video/audio data.

A new multiobjective approach to solve the routing problem in WMSNs is presented in [28]. Delay and expected transmission count were considered as QoS requirements. The authors of [28] could avoid conflicts among the QoS parameters that may lead to suboptimal solutions.

A novel routing approach named GEEC is reported in [17]. GEEC belongs to the group of clustering routing protocols. The simultaneous achievement of energy exhausts the equilibrium, and lifetime extension was gained through the mechanism of evolutionary game theory.

The work proposed in [18] is a clustering protocol for WSNs. This work can be separated into three phases. First, an algorithm for optimal cluster size was given along with a mathematical model targeting to solve the hotspot problem. Next, for the prevention of anarchism in selecting CH, the evolutionary game theory is used. Finally, a novel energy-efficient routing protocol based on evolutionary game theory is proposed.

As discussed earlier, the amount of data is large in WMSNs, data redundancy can cause a significant level of energy wastage and reduced network lifetime. So far, there are two protocols that have worked upon routing to avoid data redundancy. QMOR [14] is one of them. The authors aim at selecting and prioritizing the forwarder list to gain an energy-efficient delivery of multimedia data under specific QoS requirements. First, the protocol introduces a redundancy avoidance mechanism to avoid multimedia traffic by taking advantage of multiple sink nodes. In that study, multi-sink aware operations were integrated into an opportunistic routing framework to reduce energy consumption. QMOR exhibits significant performance increase in terms of energy consumption, delay and reliability. In QMOR, opportunistic routing is exploited; thus, overhearing occurs in the network that can significantly decrease the network lifetime because of the huge chunks of data.

A protocol proposed by Alae and Barcelo-Ordinas facilitates multimedia node clustering that satisfies FoV constraints. In this routing approach, the main criteria for creating clusters is the overlapping of FoVs of the two nearby sensor nodes. Given that the overlapped area of two multimedia sensor nodes is wide, the two sensors will act similarly from the coverage point of view, and they are selected as cluster members. It is not necessary for nodes in the same clusters to be neighbors. This clustering method conserves energy and prolongs network lifetime by creating the potential of cooperation among nodes belonging to the same cluster and avoiding redundant sensing and processing [20]. The proposed scheme does not compare the performance results with any other similar or standardized protocol. Furthermore, important routing metrics, such as packet delivery ratio or end-to-end delay, were not considered. We refer to this protocol as AB routing (Alae and Barcelo-Ordinas routing) for simplicity in our study.

LEACH is a distributed cluster formation algorithm. There are advantages of its being distributed; however, it offers no guarantee about the placement and/or number of the CH nodes. Using a central control algorithm to form the clusters may produce better clusters by dispersing the CH nodes throughout the network. This is the basis for LEACH-C, a protocol that uses a centralized clustering algorithm. The centralized scheme can be advantageous to WMSNs because any bad organization in the cluster may cause inefficient data acquisition. Readers can refer to [21] for further description of LEACH-C.

## 3. Network Model and Problem Statements

Network model and problem statements are presented in this section. Some assumptions are summarized first. We assume that every single node has enough power to transmit data to the sink nodes. We also assume that the nodes can vary their transmission power and support different established MAC protocols. We will use a model in which we always have data to transmit and the nearby nodes have co-related data. In this section of the study, we describe the network model and the problem statement for the proposed protocol. We will describe our network model further and mathematically formulate the problem statement.

### 3.1. Network Model

For our network model, we consider a 100 × 100 m network space in which multimedia sensor nodes are randomly and uniformly scattered. In the clustered sensor networks, there are three types of nodes: CH, CM, and sink nodes:

CH nodes: These nodes are responsible for communication with the sink nodes. They receive the collected data from other member nodes in the cluster and do not participate in data acquisition when they act as CHs. The role of being a CH rotates among the nodes and is selected optimally using game theoretic computations. There are limited number of CHs in a network adhering to other member nodes in the cluster that are within the range.

CM nodes: These nodes are responsible for collecting data from the network. Periodically, they report to the CHs and do not transmit data to the sink nodes directly. For our network, CMs compete for the FoV of the object and depending on their energy level, one of the two competing nodes wins and transmits the data to the CH.

Sink nodes: These nodes receive data from the CHs and have higher power level and computation capacity. Sink nodes also act as a gateway to connect to the outer world, and users may directly request data fetched from them remotely. There are three sink nodes in the network. CHs transmit data to the sink nodes based on the distance.

Multi-sink network architecture: A sensor node is usually assumed to be covered by a sink node. However, a sensor node is uncovered by a sink node if the received signal strength is below a certain threshold level. In this paper, the process of deciding the number of sink nodes is carried out iteratively. Initially, it is assumed that none of the sensor nodes is covered by a sink node and the set of the sink nodes is empty. Then, the number of the sink nodes is iteratively increased one by one so that every sensor node has dual paths to two sink nodes for providing redundant paths against sink failure. To ensure all the sensor nodes in the network to have dual paths to two different sink nodes, we found out that three sink nodes are necessary in our simulation study. It will be described in Section 5.1.

**Figure 1:**
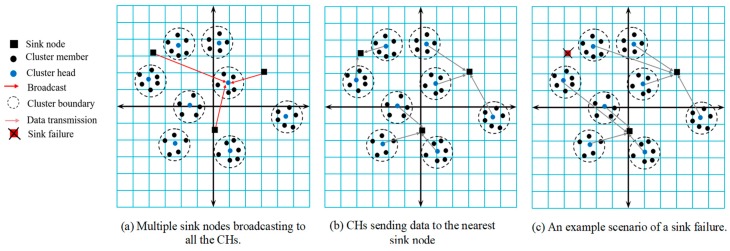
Example network scenarios.

Figure 1a–c shows the network scenarios wherein multiple sink nodes have been deployed. In Figure 1a, sink nodes broadcast to the CH nodes, and based on the RSSI value, the CH will decide which sink it should choose to transmit the collected data to. In Figure 1b, CHs in different clusters transmit data to their nearest sink node for faster and reliable data delivery. The dashed circles represent a cluster, black circular nodes represent CMs, blue circular nodes represent CHs, and quadrilaterals represent sink nodes. The role of a sink node is of utmost importance in a WMSN because the entire network is useless without it. Failure of a sink node is not desired and use of multiple sinks can increase reliability greatly. Figure 1c shows that even if a sink node fails/runs out of energy, the WMSN can complete its task.

### 3.2. Problem Statements

In this subsection, the problem is formally defined for developing a routing protocol based on EG for WMSNs with multiple sinks. The main objective of the proposed protocol lies in electing CHs using EG. The first problem statement describes this phenomenon mathematically for the convenience of readers. Another contribution of EGR is the data redundancy avoidance method to efficiently manage the large amount of data in WMSNs. The second problem statement describes this objective:

Problem statement 1: Given a set of sensor nodes in the network, *S* = {*s*_1_, *s*_2_, …, *s_n_*}, our objective is to determine the following:

- The number of clusters *k*.
- A set of CHs determined using EG, *H* = {*h*_1_, *h*_2_, …, *h_k_*}, and *k* clusters *C*_1_ = {*h*_1_}, *C*_2_ = {*h*_2_}, …, *C_k_* = {*h_k_*}. Here, H is the set of CHs and *h*_1_, *h*_2_, …, *h_k_* are the CH nodes in cluster *H*. *C*_1_, *C*_2_, …, *C_k_* present different clusters in the network.
- A set of CMs determined using EG, *M* = {*m*_1_, *m*_2_, …, *m_k_*}. Here, *M* is the set of CMs and *m*_1_, *m*_2_, …, *m_k_* are the CM nodes in a cluster.

Problem statement 2: Given that *M* = {*m*_1_, *m*_2_, …, *m_k_*} and number of sensor nodes, *n* ≤ 100, our objective is to determine the following:

- The overlapped region between two nearby sensor nodes (for example *m*_1_ and *m*_2_) to choose one from the two based on the average residual energy if the overlapped area *A*, crosses a certain threshold *T*.
- If the data captured (still images for our case) by one sensor node is identical to another based on their overlapped FoVs to avoid transmission of same type of data.

## 4. EGR Protocol

In this section, a robust routing protocol called EGR for WMSNs with multiple sinks is presented, which is based on EG. This clustering technique conserves a lot of energy in WMSNs as well as conventional WSNs because the network performance is significantly improved by decreasing the number of hops from the sensor nodes (SNs) to sink node and reducing the amount of data to be delivered [29,30]. In cluster-based networks, more energy is consumed by CH nodes than the normal SNs (which can also be regarded as cluster members). It is obvious because more computing and communication loads are assigned to CHs [31]. The variation in the energy consumed by nodes being CHs and CMs may cause unexpected early death of some nodes. LEACH is the most famous clustering protocol that can resolve this type of energy imbalance [21].

**Figure 2:**
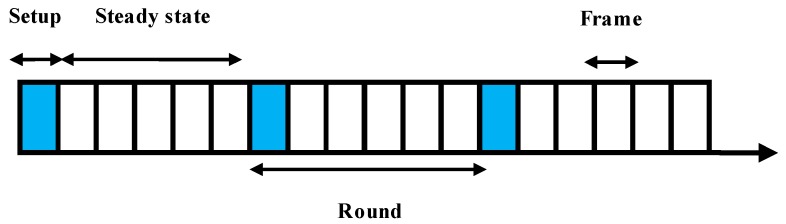
Setup and steady-state phases of EGR.

In EGR, nodes will organize themselves into clusters based on overlapped FoVs. One node will act as a CH and other CMs will transmit collected data to the CH. CHs will transmit data to sink nodes after some processing. Therefore, energy expenditure of the CH nodes is higher than that of the CM nodes. The operation of EGR protocol can be divided into rounds as discussed earlier (“setup” and “steady-state” phases). In the “setup” phase, clusters are formed, CH nodes are selected intelligently using EG, CHs advertise to the CMs within the cluster, and CMs send join requests to the CHs. In the “steady state,” CHs determine the sink to transmit data based on the RSSI value. Finally, a data redundancy avoidance algorithm is used before processing and transmitting the data to the sink nodes by CHs. Figure 2 shows the traditional rounding system used in our EGR routing protocol. Additionally, Table 2 presents the operations in two states of a round in EGR.

We will describe our entire routing process in the following sections. In Section 4.1, we will describe the cluster formation technique. In Section 4.2, we will elaborate the CH election process. In Section 4.3, we will describe our data redundancy avoidance algorithm along with the proposed EGR algorithm.

### 4.1. Cluster Formation

**Figure 3:**
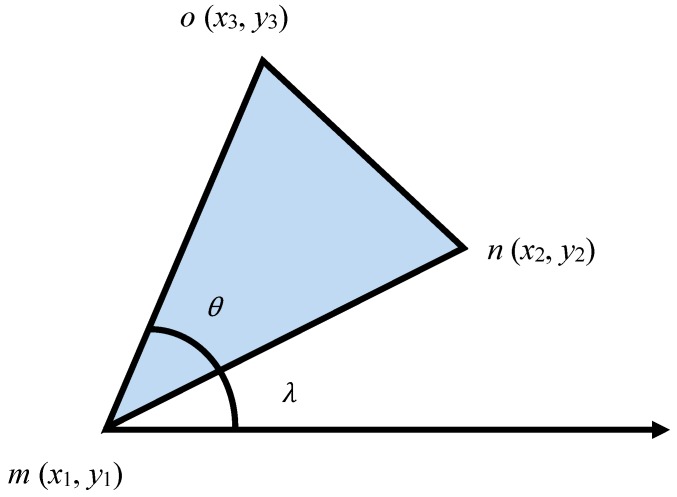
FoV of a camera in a multimedia sensor node.

Multimedia sensors, such as cameras, have directional views and can capture multimedia data based on that. Multimedia sensor nodes deployed randomly and densely with fixed lenses (having a certain angle) are assumed in this study because most of the existing WMSN platforms (such as SensEye and Panotes) have fixed lenses. It is also assumed that sensors are aware of their positions. All the sensor nodes must be equipped to have the information of their location coordinates and orientation. Any lightweight localization techniques can be used in this case. We mentioned the overlapping area earlier in this study and now would like to introduce a term threshold (*T*), which is the minimum percentage of the overlapped region for the FoVs. This threshold will be used to define the cluster formation process. It will denote the minimum percentage of the overlapped region between the sensor nodes’ FoV for membership inclusion in the cluster. Figure 3 shows the FoV of a multimedia sensor node.

In Figure 3, we can assume the FoV to be an isosceles triangle having two congruent sides. The vertex angle can be represented as theta (*θ*). The length of the congruent sides can be denoted as *mn* = *Cs*. The orientation angle is *λ*. Let us also assume a sensor that is located at point m (*x_1_, y_1_*). To determine the coordinates of “*n*” and “*o*” points, the following formulations can be undertaken:

(1) $$x_{3}=x_{1}+C_{s}\cdot \cos\left(\lambda \right).$$

(2) $$y_{3}=y_{1}+C_{s}\cdot \sin\left(\lambda \right).$$

(3) $$x_{2}=x_{1}+C_{s}\cdot \cos\left(\left(\lambda +\theta \right)mod2\pi \right).$$

(4) $$y_{2}=y_{1}+C_{s}\cdot \sin\left(\left(\lambda +\theta \right)\mathrm{mod}2\pi \right).$$

**Figure 4:**
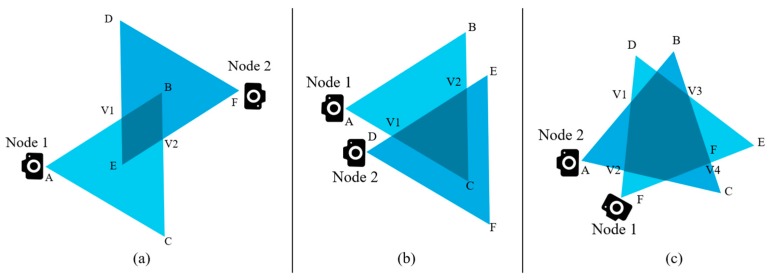
Different geometric shapes that can be caused because of the overlapped FoV: (**a**) Quadrilateral; (**b**) Triangle; (**c**) Polygon.

If we know the coordinates of a triangle, it requires only simple matrix operations to compute the area. This is necessary because a certain portion of the area (1/2 or 3/4) of the triangle is to be defined later as threshold in our cluster formation technique and data redundancy avoidance algorithm. There are three possible geometrical shapes that can be noted by observing the overlapping FoVs of two nearby multimedia sensor nodes. They are triangle, quadrilateral, and polygon. Figure 4 shows the different shapes of the overlapped area. The shaded regions represent the overlapped area in the figure.

The threshold *T* can be defined as half of the area of the triangle created because of the FoV of a sensor node. The nodes that are close to each other, among which the overlapped FoV is greater than the threshold, can be in the same cluster. For example, the nodes shown in Figure 4a cannot belong to the same cluster; however, nodes in Figure 4c may belong to the same cluster. For EGR, the algorithm to form the cluster is executed in a centralized way via sink nodes. This centralized architecture was used because of the following reasons:

- If the architecture is distributed, each node must notify the rest of the nodes about its location and orientation.
- The sink nodes can be provided with adequate amount of resources, such as storage and power supply. The normal nodes are more resource constrained; thus, sink nodes can play this role better.
- Information collection through the sink node is more power efficient than collecting and spreading this information to each node in the network.
- Our cluster election process using EG requires nodes in the clusters to be divided based on their remaining energy. This is performed using the sink node.
- Finally, using a centralized scheme, it is possible to relieve the processing load from the sensors and network lifetime.

**Figure 5:**
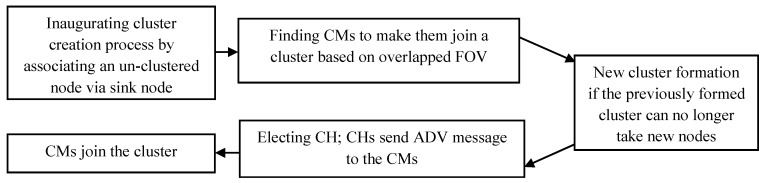
Cluster formation process in EGR.

Figure 5 shows the cluster formation procedure step by step for better understanding.

**Figure 6:**
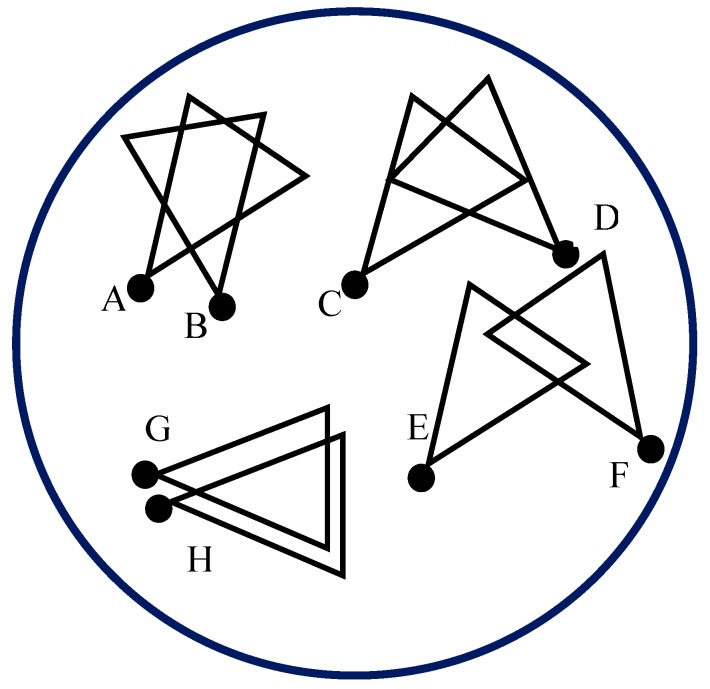
Overlapped FoVs of sensor nodes in a network area.

To form a cluster, first, the sink creates an empty cluster by associating an un-clustered sensor node. The node will be the first member in the cluster. Subsequently, the sink finds the CMs by computing the overlapped area and comparing it with the threshold. For example, if the triangle created by the FoV of a sensor node has an area of 92 units, the threshold value *T* can be set to the half of the area, which is 46 units. Any two sensor nodes having overlapped FoV more than that value would fall into the category of being in the same cluster. Figure 6 shows the overlapped FoVs of some sensor nodes. Nodes *A* and *B* will be in the same cluster because of their overlapped FoV crossing the threshold *T*. This is also true for nodes G and H. Not only nodes C and D but also nodes E and F will not be in the same cluster for their overlapped FoV being smaller than threshold *T*.

**Figure 7:**
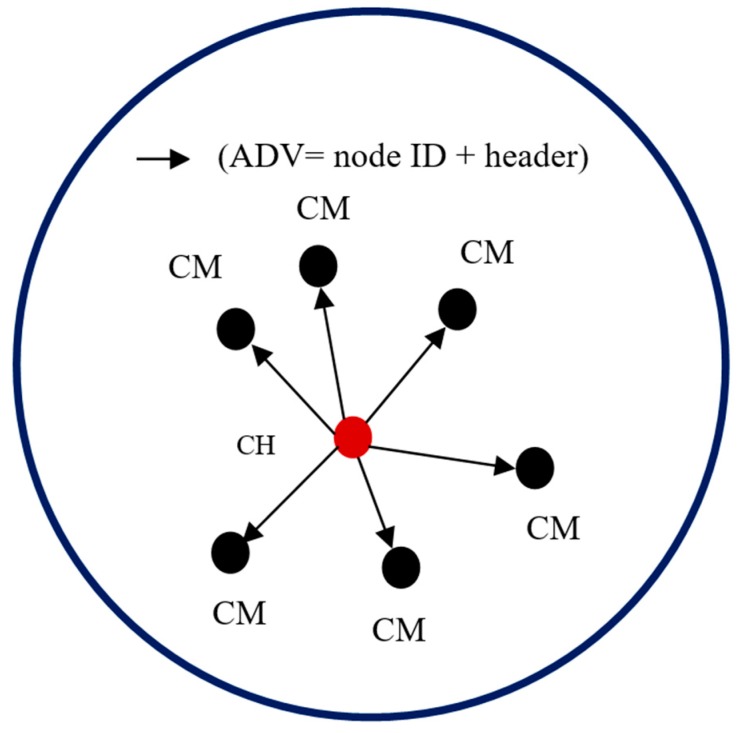
CH advertising to the CMs.

**Figure 8:**
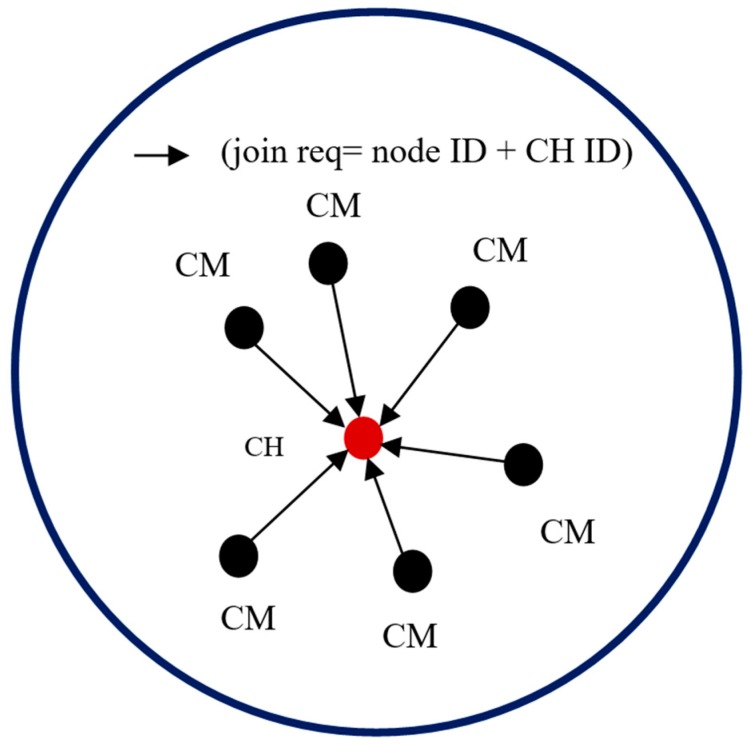
CMs sending join request to the CH.

When no more nodes can be added to the cluster, the sink takes a new un-clustered node and begins the cluster formation process again. Each CH that is elected using EG must broadcast an advertisement (ADV) message. The message is segmented into the node’s ID and a header that makes this message distinguishable as an announcement message. When the CMs determine the clusters they will join, they must inform the elected CHs about their membership in the clusters. Each CM node sends a join request to a CH. The join request message contains the “node ID” and “CH ID.” If a node receives more than one ADV message, it joins the CH with the highest energy. In exceptional cases, a CM node may not receive an ADV message. In this case, it can either keep itself aloof from routing or declare itself as a CH based on the remaining energy it possesses. Figure 7 and Figure 8 illustrate the process of a CH broadcasting to CMs and the process of CMs informing a CH about their membership, respectively.

### 4.2. CH Election Process Using EG Theory 

In this section, we elaborately discuss the process of optimal CH election using EG. Being a CH decreases the energy level more than being CM. Being a CM will increase the lifetime of a node. Being a CH decreases the lifetime of a node; however, if all nodes decide to remain CM, the network will lose the cluster-based functionalities. The EG designed and formulated to elect CHs had three essential components. It is modeled as: EG (*P, S, U*), where *P*, *S*, and *U* represent a set of players, set of strategies, and utility function, respectively. *P* consists of sensor nodes that act as players of the game. *P* = {*p*_1_, *p*_2_, *p*_3_, *…*, *p_n_*}; where *p*_1_, *p*_2_, *p*_3_, *…, p_n_* are sensor nodes in the cluster. These nodes in set *P* are classified into two subsets:

(5) P=NH∪NL,

where *NH* is a set of nodes that have higher level of residual energy as compared to a certain energy threshold (*E_T_*), *NL* is a set of nodes that have lower remaining energy as compared to *E_T_*, *NH*⊂*P*, and *NL*⊂*P*. The strategy set *S* has two elements:

(6) $$S=\left\{T_{CH},T_{NCH}\right\},$$

where *T_CH_* represents the strategy of being a CH and *T_NCH_* represents the strategy of not being a CH. As mentioned earlier, *U* presents the utility function. The basic function to calculate the utilities can be presented by the following equation:

(7) $$U_{i}=R_{i}-P_{i},$$

where *i* represents the node we are calculating the utility of, and *R_i_* and *P_i_* are the reward and penalty for node *i*, respectively, based on the action it chooses while playing the game. Using Equation (7), the complex utility functions for nodes falling under *NH* and *NL* sets will be formulated. In EG, formulating the evolutionary stable strategy (ESS) is an important step. The ESS ensures that different species in a population co-exist together and do not threaten each other by increasing the extinction probability through selfish choice of strategies. For the game designed in our protocol EGR, ESS for CH election game is *T_CH_* for nodes in the *NH* subset and *T_NCH_* for nodes in the *NL* subset. 

If the rate of nodes in set *NH* to become CHs (selecting the $T_{CH}$ strategy) is ρ, the rate of them to become CMs (selecting the $T_{NCH}$ strategy) is (1−ρ). This condition is also true for nodes in set *NL*. Therefore, the rate of nodes in set *NL* to become CHs (selecting the $T_{CH}$ strategy) is *η* and the rate of them to become CMs (selecting the $T_{NCH}$ strategy) is (1−η). Based on these assumptions, the expected utility for nodes in set *NH* to become CHs by selecting the $T_{CH}$ strategy is expressed as follows:

(8) $$U_{NH\left(CH\right)}=\eta \alpha +\left(1-\eta \right)\left(\alpha +2\delta \right)=\alpha +2\delta -2\delta \eta ,$$

The utility of nodes in *NH* to become CMs by selecting the $T_{NCH}$ strategy is:

(9) $$U_{NH\left(NCH\right)}=\eta \left(\alpha -\delta \right)+\left(1-\eta \right)\left(-\delta \right)=\eta \alpha -\eta \delta -\delta +\eta \delta =\eta \alpha -\delta .$$

Therefore, the total revenue for nodes in *NH* is expressed as:

(10) $$\bar{U_{NH}}=\rho \left(\alpha +2\delta -2\delta \eta \right)+\left(1-\rho \right)\left(\eta \alpha -\delta \right).$$

In EG, the change of rate of players’ action is called replicator dynamics. After mathematical analysis, the replicator dynamic equation for nodes in *NH* is obtained by the following equation:

(11) $$F\left(\rho \right)=\frac{d\rho }{dt}=\rho \left[U_{NH\left(CH\right)}-\bar{U_{NH}}\right]=\rho \left(1-\rho \right)\left[\alpha +3\delta -\eta \left(2\delta -\alpha \right)\right].$$

Similarly, the equations to calculate the utility for nodes in *NL* is formulated. Equations (12) and (13) present the utility of nodes in *NL* to become CHs by choosing $T_{CH}$ strategy and the utility of nodes in *NL* set to become CMs by choosing the $T_{NCH}$ strategy, respectively:

(12) $$U_{NL\left(CH\right)}=\rho \beta +\left(1-\rho \right)\left(\beta +\delta \right)=\beta +\delta -\rho \delta .$$

(13) $$U_{NL\left(NCH\right)}=\rho \left(\beta +2\delta \right)+\left(1-\rho \right)\left(-\delta \right)=\beta \rho +3\rho \delta -\delta .$$

Therefore, the total revenue for nodes in set *NL* is expressed as:

(14) $$\bar{U_{NL}}=\eta \left(\beta +\delta -\rho \delta \right)+\left(1-\eta \right)\left(\beta \rho +3\rho \delta -\delta \right).$$

The replicator dynamic equation for nodes in *NL* set is formulated as:

(15) $$\frac{d\eta }{dt}=H\left(\eta \right)=\eta \left[U_{NL\left(CH\right)}-\bar{U_{NL}}\right]=\eta \left(1-\eta \right)\left[\beta +2\delta -\left(\beta +4\delta \right)\rho \right].$$

**Figure 9:**
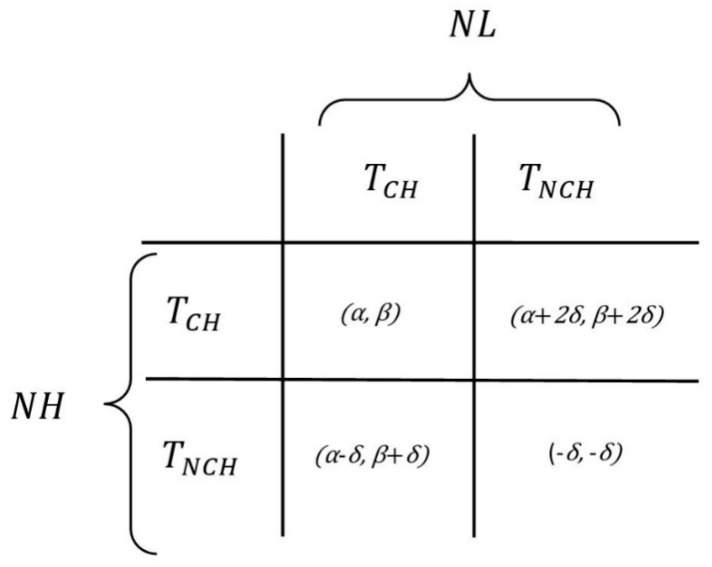
Utility for choosing variant strategies in the designed game.

Figure 9 shows the utility matrix for the formulated game. It shows the amount of reward a node in *NH* and *NL* receives by selecting either $T_{CH}$ or $T_{NCH}$ strategy.

When the nodes get divided into clusters, it is necessary to divide them into *NH* and *NL*. All the nodes in different clusters send an initial message to the sink node. This message contains two information. One is the node ID, and another is the residual energy level of the node, *N_RE_*. The sink node segregates the nodes based on the *N_RE_* of the node. A median value is selected as *E_T_*, which represents the energy threshold. The sink node broadcasts this value to the nodes in different clusters. All the nodes in the clusters divide themselves into either *NL* or *NH* based on the threshold value. After that, utilities are calculated using Equations (10) and (14) presented earlier. The nodes either decide to be CH or CM by choosing the $T_{CH}$ or $T_{NCH}$ strategies based on the calculated utilities.

### 4.3. Data Redundancy Avoidance and EGR Routing Algorithm

**Figure 10:**
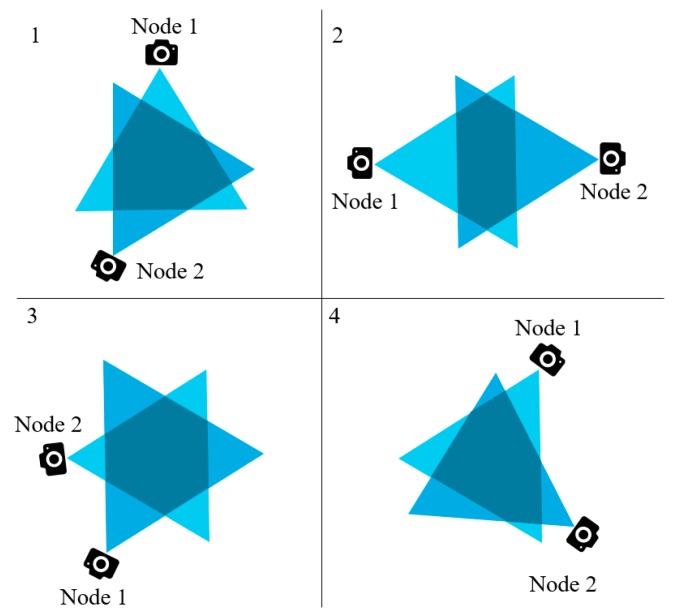
Different types of polygons created by the overlapped FoVs of two closely placed sensor nodes: (**1**) sensor nodes placed vertically opposite to each other; (**2**) sensor nodes placed horizontally opposite to each other; (**3**) Sensor nodes placed closely to the left side of their orientation; (**4**) Sensor nodes placed closely to the right side of their orientation.

In the previous sections, we discussed the overlapped regions of FoV created because of the closely placed multimedia sensor nodes. We also introduced the term threshold *T*, which is a certain portion (for our case, we assume it to be half) of the isosceles triangle of the FoV. The congruent sides of the triangle are denoted by C_s_. According to our observations, if any object falls in the FoV of two closely placed sensor nodes and the FoVs of them creates a polygon, the data will be redundant if both nodes send the snapshot of the object. If any object falls within the shaded region shown in Figure 10, the FoVs for that object to node 1 and node 2 are almost similar. 

**Figure 11:**
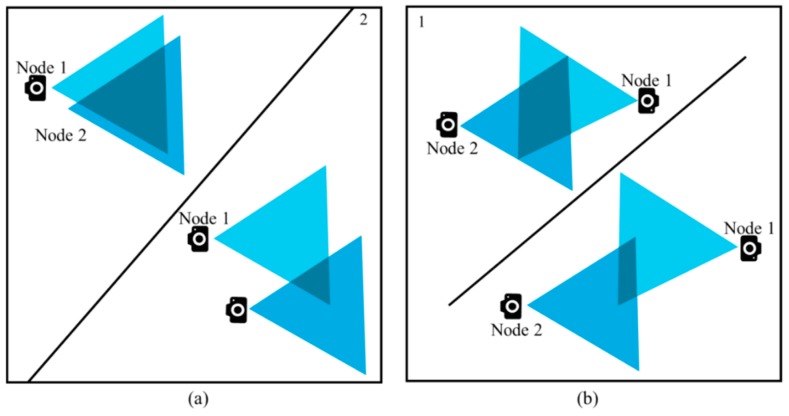
Different sizes of (**a**) triangles and (**b**) quadrilaterals created because of the overlapped FoVs.

For geometric shapes like triangle and quadrilateral, we cannot be sure if the overlapped area is big enough to cross the threshold value (*T*), or small enough to be under the threshold. The triangles or quadrilaterals created because of the overlapped FoVs can be small or big. Figure 11a,b shows such kind of observations. Based on all these assumptions and interpretations, our data redundancy avoidance algorithm is as follows:

**Algorithm 1** Data redundancy avoidance1. Node 1 **→**
*n*_1_, Node 2 **→**
*n*_2_ 2. Residual energy of *n*_1_**→**
*R*_1_, residual energy of *n*_2_**→**
*R*_2_ 3. Initialize threshold = *T* 4. Check if the Euclidian distance between two nodes, *D >* 2*C_s_* 5. **if** (*D* > 2*C_s_*) 6.   No overlapping FoV exists 7. **end if** 8. **if** (*D ≤ C_s_*) 9.   **if** geometrical shape is polygon 10.     data is redundant 11.     **end if** 12.      **if** geometrical shape is quadrilateral **||** triangle 13.     compute the area of the quadrilateral triangle 14.     **end if** 15.      **if** (Area > *T*) 16.       data is redundant 17.      **else**
18.       data is not redundant 19.      **end if** 20. **end if** 21. **if** (*R*_1_
*> R*_2_) 22.  *n*_1_ will own the object’s FoV and transmit to CH accordingly 23. **else** 24.  *n*_2_ will own the object’s FoV and transmit to CH accordingly 25. **end if**

**Figure 12:**
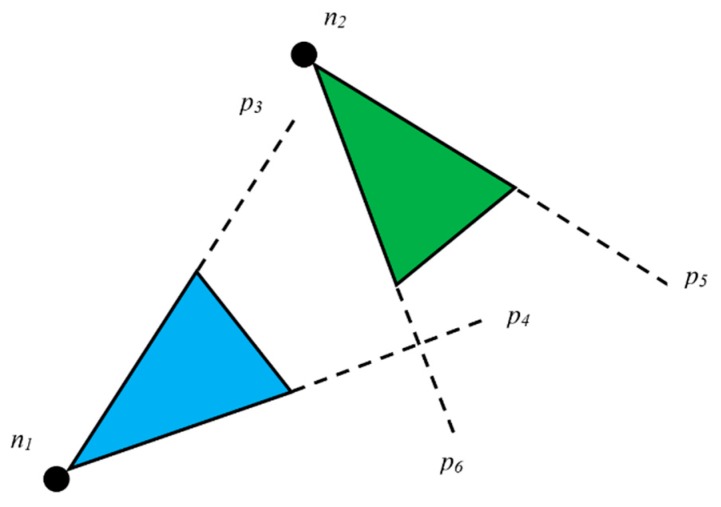
Scenario where two nodes have a distance that is two times more than the congruent sides of the triangles created due to FoV.

Algorithm 1 basically presents how redundant data between two nodes are dealt with. First, two nodes ofn_1_ and n_2_ are initialized. R_1_ and R_2_ are declared as residual energy levels of n_1_ and n_2_, respectively. We discussed the area threshold T earlier in Section 4.1 while discussing the cluster formation procedure. The threshold is also needed in this algorithm. If the distance between the two nodes is two times greater than the congruent sides of the FoV created by them, it is impossible to have a FoV overlap. Figure 12 presents a scenario where two nodes have a distance that is two times more than the congruent sides of the triangles created due to FoV.

If the case is not as in Figure 12, we can conclude that there is a possibility of data redundancy. In this algorithm, we consider data to be redundant if the area created by the two FoVs overlaps to form a polygon. If it forms either a triangle or a quadrilateral, we must compute its area and compare it with the threshold to determine if the data is redundant.

**Figure 13:**
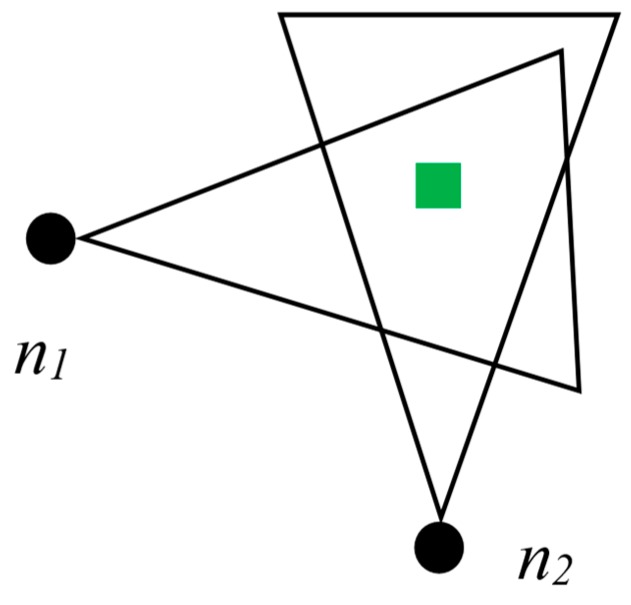
A scenario where two nodes have a distance that is less than two times of the congruent sides of the triangles created due to FoV.

If a view of an object is observed to be same for two nodes, the node with higher energy will win the FoV and transmit the data to CH. In Figure 13, from two nodes ofn_1_ and n_2_, the one that has higher residual energy will win the FoV of the object that is represented as a rectangular box in the figure.

**Figure 14:**
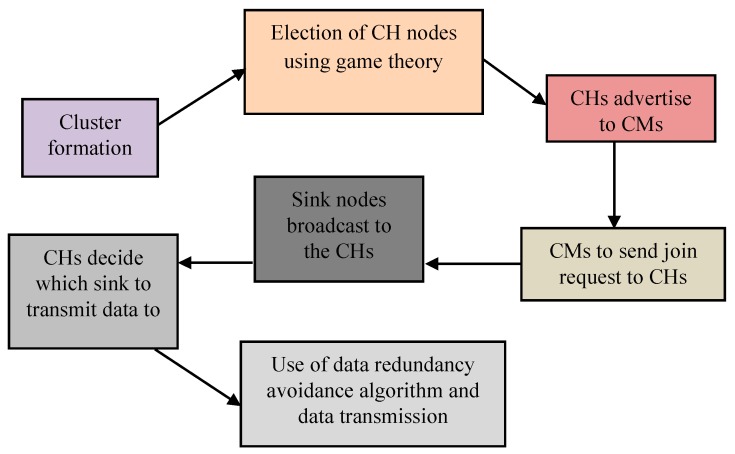
Overall process for routing in the proposed EGR.

The data redundancy avoidance algorithm and optimal CH selection procedure using EG are the core mechanisms of the proposed EGR routing protocol. The entire routing process can be viewed in Figure 14:

EGR’s architecture is similar to that of LEACH; however, the core differences are: (1) cluster formation using the FoVs of the multimedia sensor nodes, (2) data redundancy avoidance using the overlapped FoVs of the sensors, and (3) optimal CH election using EG. The algorithm for our routing process is as follows:

**Algorithm 2** Routing Algorithm1. Initialize *S* to be the set of nodes (*S* = {*S*_1_, *S*_2_, *S*_3_, …, *S_n_*}) 2. Highest number of nodes to be in cluster → *Q* 3. Number of rounds → *I_n_* 4. Final round → *I_f_* 5. Threshold area → *T* 6. *T* = ½ (Area of the isosceles triangle created by FoV) 7. **loop** until every single node is put at least into one cluster 8.     Empty cluster creation by associating the un-clustered node 9.     *S_n_* → First member of the cluster 10.   Overlapped area computation between *S_n_* and *S_n_* ± *m* 11.   **while** (number of nodes in the cluster < *Q*) 12.       **if** (Area calculated > *T*) 13.        *S_n_ ± m* is added to the cluster (*m* = any integer) 14.       **else** 15.        Consider another node for cluster formation 16.   **end while** 17. **end loop** 18. Decide the CHs using EG 19. Advertise as CHs to the CMs within the cluster **Steady-state phase:**
20. Sink nodes broadcast to the CHs. 21. CHs decide which sink to transmit based on the RSSI value. 22. Avoidance of data redundancy using Algorithm 1. 23. CMs send data to the CHs. 24. CHs deliver data to the nearest sink node. 25. Repeat the whole process until final round.

In Algorithm 2, a set of nodes that will participate in routing are initialized. Also, we want to put a certain limit to the number of nodes in a cluster. For that, *Q* is initialized as the number of CMs that can remain in a cluster. Nodes are included into a cluster until they reach *Q*. As stated earlier, the operational architecture of EGR is similar to that of LEACH, including the rounding mechanism. *I_n_* and *I_f_* have been initialized as the number of rounds and the final round, respectively. The routing process is repeatedly executed through the setup and steady-state phases till the final round. In the setup phase, clusters are formed (see Section 4.1 for more details), and CHs are elected using EG (see Section 4.2 for more details). The steady-state phase is longer in time duration than the setup phase. Sink nodes broadcast to CHs in the steady-state phase, and CHs decide the sink node based on RSSI value. At this point, Algorithm 1 is used to get rid of redundant data. Then, CMs send data to their CHs, and CHs deliver data to the sink node.

As mentioned earlier, Algorithm 1 is used in the main routing algorithm of Algorithm 2. Therefore, we analyze Algorithm 2 rigorously. The time complexity of the proposed routing algorithm can be calculated as:

(16) $$O\left(1\right)\;+\;O\left(n\right)\;+\;O\left(2mn\right)\;+\;O\left(Hn\right)\;+\;O\left(H\right)\;+\;O\left(p\left(p\;-\;q\right)\right)+\;O\left(M\right)+\;O\left(3Y\right)\;+\;O\left(3Y\right)\;+\;O\left(n\right)\;+\;O\left(HM\right)\;+\;O\left(M\right)\;+\;O\left(n\right)\;=\;O\left(n^{2}\right)$$

where *n* is the total number of nodes in the network and *Y* ≤ *M* ≤ *H* ≤ *n*. The complexity is quadratic time because of the nested loops and the use of two-dimensional arrays are included in the algorithm while implementing pay-off matrix for game-theoretic formulation.

## 5. Performance Evaluation

In this section, the performance of the proposed EGR is evaluated via computer simulation and then compared to those obtained using conventional protocols. We compare the proposed EGR with GEEC, EEREG, QMOR, AB routing [20], and LEACH-C [21]. Similar to EGR, QMOR considers multiple sinks and data redundancy avoidance. AB routing have clustering mechanism along with data redundancy avoidance in WMSNs. LEACH-C is a widely used clustering-based protocol for centralized WSNs, which is selected for performance study and for investigating how our proposed protocol performs against a real-time protocol that is commonly used in the industry. For simulation, the experimental environment for wireless multimedia sensor networks built upon Castalia/Omnet++ called WISE-Mnet++ is used.

### 5.1. Simulation Environment

To evaluate the performance of our EGR protocol in the mentioned simulation environment, it is necessary to adopt some performance metrics. The quality of a routing protocol is indicated by the performance metrics [32]. We will consider five performance metrics to compare the performance of EGR with the existing works. They are average residual energy, average end-to-end delay, network lifetime, average packet delivery ratio, and cluster formation time. The performance metrics are defined as follows:

- Average residual energy is the average remaining energy of a node after a certain amount of time in simulation.
- Network lifetime is the elapsed time until half of the nodes are alive.
- Average end-to-end delay is the average time required for a packet to reach its destination from source.
- Average packet delivery ratio is the ratio of the received packets over packets sent in the network.
- Cluster formation time is the time taken to form all clusters in the network.

The simulation environment consists of 100 × 100 m network space. For the simulation, we consider a random deployment of 100 multimedia sensor nodes, and there are three sinks nodes in the network. The environment consists of a moving target. Still images are captured by the nodes who win FoVs of the target component. Each node has an initial energy of 2 J and can sense up to 30 m. Table 3 shows the simulation environment for the deployment of the proposed protocol. Table 4 shows the energy expenditure of a multimedia node for some basic operations.

In Table 5, the workload parameters of our simulation are summarized. Not only the traffic load including packet outgoing rate, packet size, and transmission rate but also the radio transmission rage is shown for the network workload incurred during the simulation. Note here that the radio transmission rage is also included in the table because it also affects the workload in accordance with interference.

Apart from the simulation results obtained using different performance metrics, we also show the effectiveness of the data redundancy avoidance algorithm (Algorithm 1) in EGR through simulation in the following section.

### 5.2. Simulation Results and Discussions

**Figure 15:**
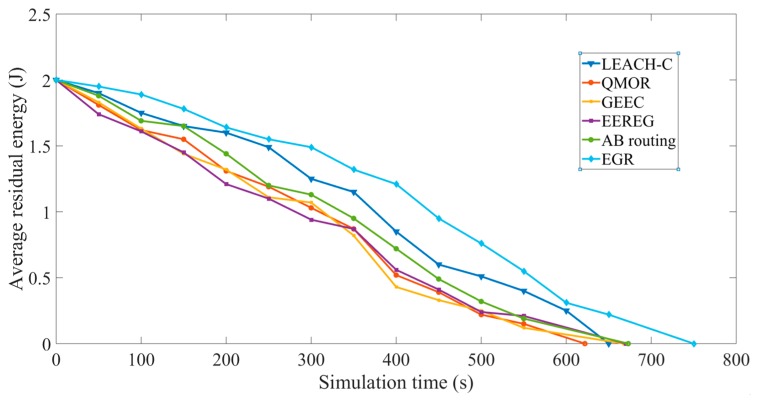
Average residual energy.

Average residual energy: Figure 15 shows the decrease of average residual energy of the sensor nodes in different protocols. The *X* axis represents the simulation time in seconds and the *Y* axis represents the average residual energy. 

As shown in Figure 15, it can be seen that the energy depleted faster in QMOR than in LEACH-C. This is because QMOR is mainly based on opportunistic routing and maintains a forwarding list of nodes capable of transmitting the data. In QMOR, a set of nodes is selected as potential forwarder. The forwarding candidates that receive the transmitted packet coordinate among each other to decide which of them must forward the packet and which must discard it. This phenomenon is mainly responsible for faster energy depletion. The energy consumption for data both received and transmitted were summed and averaged in each round to calculate the average residual energy. LEACH-C is a cluster-based routing protocol, and there is no such problem of selecting candidate forwarding nodes among which some may even drop data packets. Furthermore, for certain frames, the data is sent to the sink node directly in QMOR which is not energy efficient. Moreover, AB routing uses a simple mechanism of clustering based on the FoVs of the nodes and redundant transmissions are avoided. The protocol is clustering based; thus, number of transmissions are reduced, and it performs better than LEACH-C because of the reduction in redundant transmission avoidance mechanism. Even though GEEC and EEREG has CH election mechanism, they do not provide any kind of data redundancy avoidance technique. EGR, the proposed protocol in this study performs better than the all these five algorithms. The obvious reasons behind this are the inclusion of a more efficient redundancy avoidance algorithm, optimal CH selection using game theory, and inclusion of multiple sink nodes that helped nodes to avoid long distance transmissions. Furthermore, it performs better than AB routing because of the optimal CH election process using EG. If the residual energy of any node *i* is *REi* at a time *t*, the average residual energy (for 100 nodes) is calculated using the following equation:

(17) $$\mathrm{Average}\;\mathrm{residual}\;\mathrm{energy}\;\left(t\right)=\frac{\sum_{i=1}^{100}RE_{i}}{100}.$$

**Figure 16:**
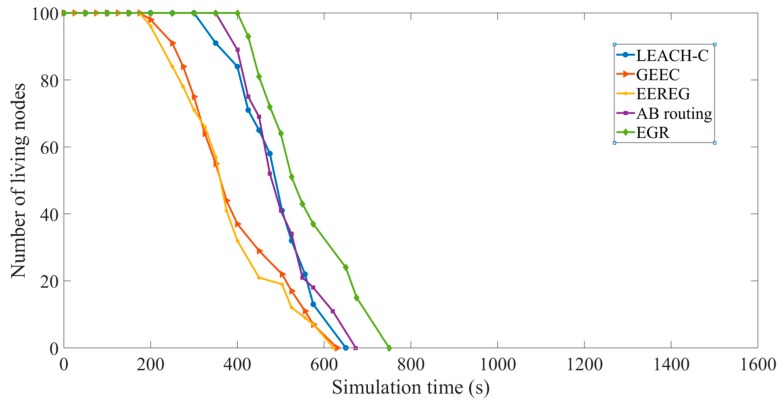
Number of living nodes showing network lifetime.

Network lifetime: Figure 16 shows a comparative graph in terms of the number of living nodes during simulation. The *X* axis represents the simulation time and the *Y* axis represents the number of living nodes in the simulation time. AB routing performs better than LEACH-C in terms of network lifetime because it has a cluster formation process based on FoV. As mentioned earlier, the network lifetime is considered as the time when half of the nodes are dead. We can see that EGR performs better than the other four protocols when the number of living nodes is 50. The reasons behind this can be summarized as below:

- EGR uses a unique CH election mechanism based on EG that allows nodes with higher residual energy level to become CHs, resulting in improved energy balance in the network.
- Transmission distance from CHs to the sink nodes in EGR becomes shorter in comparison to the other protocols.
- Data volume and the number of transmissions have been reduced in EGR.

**Figure 17:**
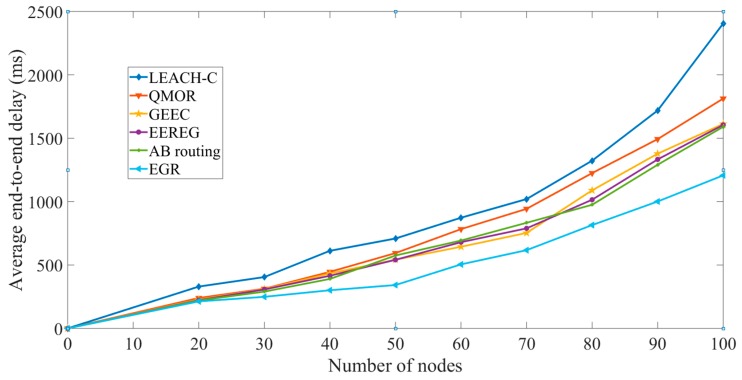
Average end-to-end delay vs. the number of nodes.

Average end-to-end delay versus the number of nodes: The simulation results were obtained for EGR in terms of end-to-end delay as well. The number of nodes was increased in different rounds and the average end-to-end delay was observed. The delay is lesser in QMOR than that in LEACH-C. The main reason for this is the direct transmission of certain data packets from source node to sink node. Furthermore, once the priority-based forwarding list is established in QMOR, the delay decreases. EGR performs better than both LEACH-C and QMOR. Also, the data volume to be delivered is reduced in EGR due to Algorithm 1, achieving lesser delay. The biggest advantage of EGR over QMOR is the reduced number of hops. EGR’s clustering architecture allowed only two hop transmissions to the closest possible sink node whereas QMOR being an opportunistic routing protocol has to undergo through multiple hops many times incurring higher delay. EGR is implemented in a scenario where nodes are involved in a moving object tracking. As time passes by, the movement of the object is increased so that the nodes could have frequent FoVs of the object. The goal behind this is to check how the data redundancy avoidance algorithm facilitates EGR with the lessened amount of multimedia data. Since GEEC and EEREG do not have any redundant data avoidance mechanism, they has to deal with higher data volume compared to EGR. Therefore, EGR has lesser end-to-end delay. Lastly, even though AB routing had a data redundancy avoidance mechanism, EGR shows better performance because of the multiple sink nodes. As shown in Figure 17, we can see that EGR performs better and shows less amount of delay than other five protocols. In summary, the use of multiple sink nodes, direct one hop transmission from the CHs to the closest sink nodes, and data redundancy avoidance mechanism are the main reasons for the performance improvement.

**Figure 18:**
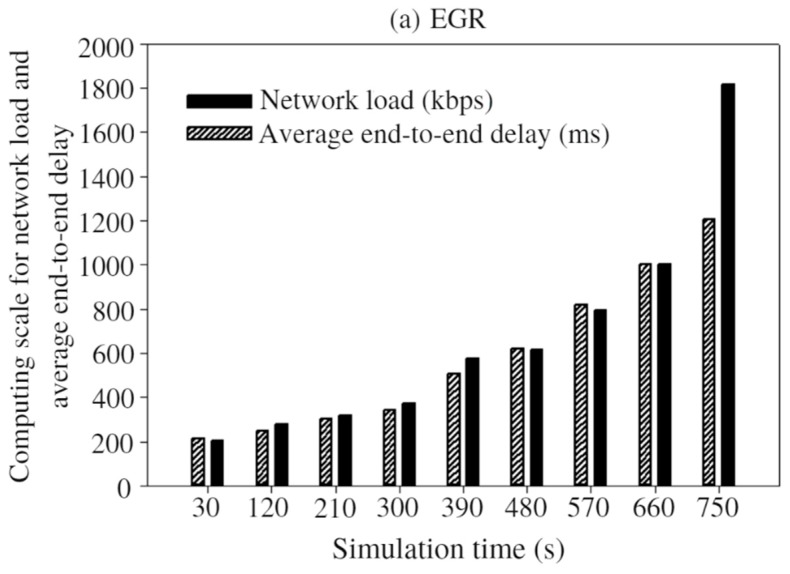

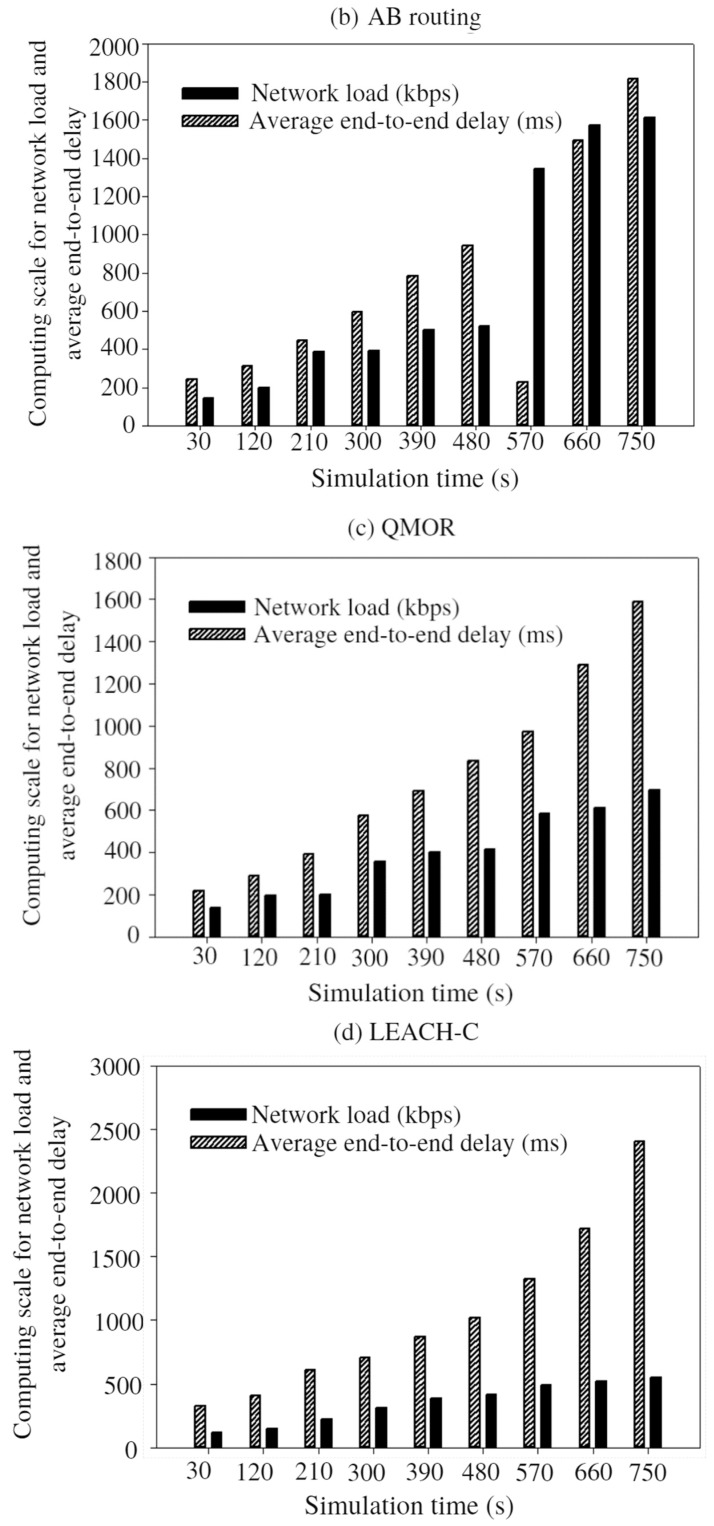

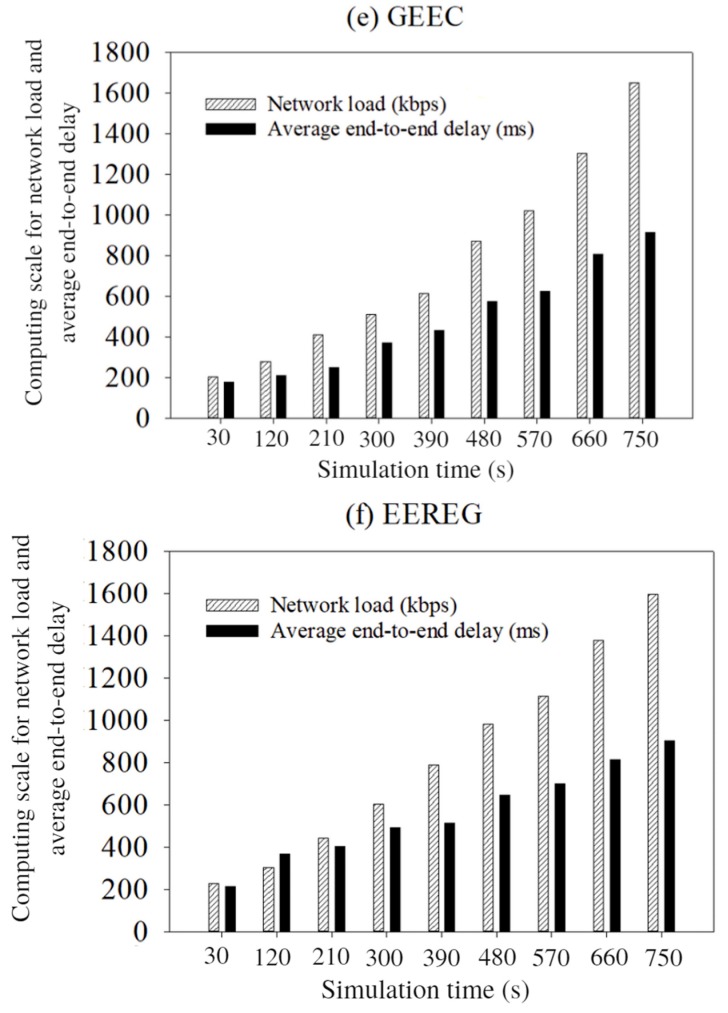
Average end-to-end delay vs. network load: (**a**) EGR; (**b**) AB routing; (**c**) QMOR; (**d**) LEACH-C; (**e**) GEEC; (**f**) EEREG.

Average end-to-end delay versus the network load: Normally, WMSNs have to deal with high volume of multimedia data. To prove the feasibility of the proposed EGR, the end-to-end delay and network load were comparatively observed along with different simulation time, as shown in Figure 18. As time increases, both end-to-end delay and network load are increased. 

In Figure 18a–c, average end-to-end delay is calculated almost in every 90 s during the whole simulation time. By observing the figures, it can be seen that EGR took 1.2 s to transmit 1.8 megabyte (MB) of data. On the other hand, QMOR, AB routing, LEACH-C, GEEC, and EEREG take the same amount of time to transmit 1.3 MB, 0.612 MB, 0.583, 0.6 MB, and 0.9 MB of data, respectively. This is mainly because of the shorter distance between the CH nodes and sink nodes in the network.

Packet delivery ratio: For this routing metric, we considered transmissions of the CHs to the sink node/s. To calculate the average packet delivery ratio, we first summed the number of data packets transmitted by each CH. Assuming that there are *N* number of clusters in the network, we determined the average number of packets transmitted using the following equation:

(18) $$Tx_{avg}=\frac{\sum_{i=1}^{N}DT_{CH_{i}}}{N},$$

where $Tx_{avg}$ is the average number of packets transmitted to the sink node and $DT_{CH_{i}}$ is the data packets transmitted by any CH in the network. Finally, the average packet delivery ratio is calculated using Equation (19):

(19) $$PDR_{avg}=\frac{Tx_{avg}}{Rx_{sink}},$$

where, $PDR_{avg}$ is the average packet delivery ratio, $Rx_{sink}$ is the data received by the sink node. Since there are multiple sinks in EGR, Equation (18) is not applicable for it. The $PDR_{avg}$ for EGR is calculated as;

(20) $$PDR_{avg}=\frac{Tx_{avg}}{\left(\frac{Rx_{sink1}+Rx_{sink2\;}+Rx_{sink3}}{3}\right)},$$

where, $Rx_{sink1},Rx_{sink2\;}$, and $Rx_{sink3}$ are the data received by the three sink nodes used in EGR.

**Figure 19:**
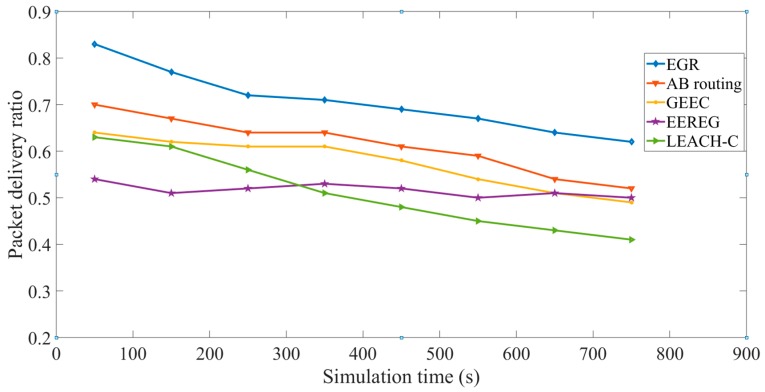
Packet delivery ratio.

QMOR is an opportunistic routing protocol. Moreover, EGR, AB routing, and LEACH-C are cluster-based routing protocols. It is not feasible to compare an opportunistic routing protocol with cluster-based routing protocols because the packet delivery ratio will always be high for opportunistic routing. Thus, we have excluded QMOR from the performance comparison for the metric of packet delivery ratio. We have calculated the average packet delivery ratio in every 100 s in the simulation time and plotted the graphs for EGR, AB routing, GEEC, EEREG, and LEACH-C. There are multiple sink nodes in EGR; thus, congestion probability is very low in it. EGR had the highest average packet delivery ratio as compared to all the protocols. Figure 19 shows the performance comparison of the five protocols in terms of average packet delivery ratio. The *X* axis represents the simulation time and the *Y* axis represents the average packet delivery ratio. As shown in this figure, we can observe that EGR outperforms the other four protocols.

Even though AB routing and LEACH-C are centrally managed, they do not have any mechanism to select CHs in the network. Therefore, the probability of low energy nodes to become CHs in the network is higher in LEACH-C and AB routing, which eventually may disrupt data transmission causing low data delivery ratio. EGR’s biggest advantage is its data redundancy avoidance algorithm along with the multiple sink nodes that not only reduce the redundant transmission of data but also decreases the congestion probability in a great manner. Therefore, it could easily outsmart GEEC and EEREG which do not have any redundancy avoidance mechanism or multisink network architecture.

**Figure 20:**
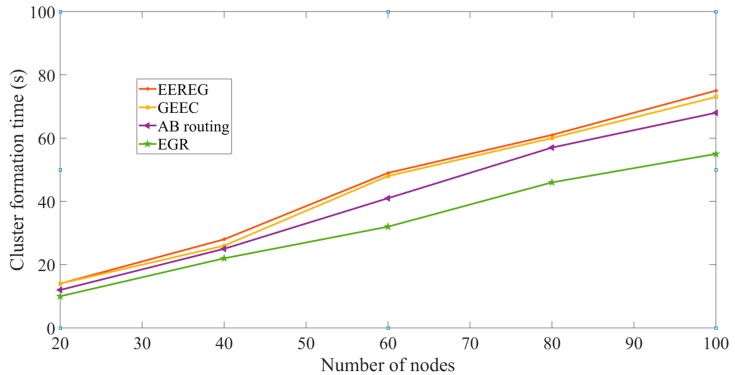
Cluster formation time.

Cluster formation time: If a long time is taken to form clusters, energy consumption can be higher along with network delay. Since WMSNs have stringent requirement for delay and they are formed with energy-hungry devices, it is necessary that the cluster formation takes less amount of time. In Figure 20, *X* axis presents the number of nodes and *Y* axis presents the cluster formation time in seconds. Cluster formation time is increased as the number of nodes increases for all the protocols. This is obvious because increasing the number of nodes would yield more clusters. As shown in Figure 20, EGR takes lesser amount of time to form clusters compared to other protocols. EGR and AB routing form clusters based on the area calculation of the overlapped FoV. EGR takes lesser time than AB routing because EGR can immediately decide to put two nodes in the same cluster, given that the overlapped FoV is a polygon. Both GEEC and EEREG have one sink node at the center of the network area. Since the whole network is managed centrally by one sink node for GEEC and EEREG, the cluster formation is delayed. On the contrary, EGR could exploit its multisink architecture to build clusters faster.

Effectiveness of the data redundancy avoidance algorithm: To prove the effectiveness of the proposed data redundancy avoidance algorithm, we have calculated the network overhead varying the number of nodes in the network. 

**Figure 21:**
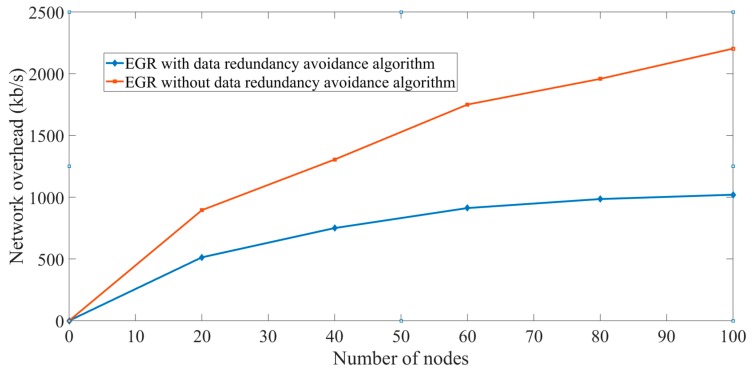
Overhead in EGR implementations with and without Algorithm 1.

In Figure 21, *X* axis represents the number of nodes and *Y* axis represents the network overhead in kB/s. First, EGR was implemented without Algorithm 1 and network load was computed by varying the number of nodes starting from 10. In this case, we can observe in Figure 21 that the network overhead is 2205 kB/s for 100 nodes. On the other hand, the network overhead is 1020 kB/s when EGR was implemented with the data redundancy avoidance algorithm. The main reason behind the overhead reduction is the implementation of the redundancy avoidance algorithm that does not allow nodes to capture and transmit the images that are identical to two closely placed nodes. This also reduces the number of transmissions significantly.

## 6. Conclusions 

In WMSNs, energy efficiency is an important design objective because the energy should support delivering large amounts of data while meeting a certain end-to-end delay requirement. This type of problem makes designing a routing protocol for WMSNs more challenging. In this study, we have proposed a new routing protocol for WMSNs, which is based on clustering technique using EG. The proposed EGR overcomes this challenge by introducing a data redundancy avoidance algorithm and intelligent CH election using EG in WMSNs with multiple sinks. Our extensive performance study shows that the EGR protocol achieves lower energy consumption, longer network lifetime, shorter end-to-end delay, and higher packet delivery ratio as compared to those in GEEC, EEREG, QMOR, AB routing, and LEACH-C. In summary, it can be concluded that EGR is more suitable for WMSNs that facilitate surveillance systems subjected to stringent QoS requirements. Furthermore, EGR can be deployed for environmental and battlefield monitoring, wherein faster data delivery is required.

## Figures and Tables

**Table 1:** Symbols and their meaning.

Symbol	Meaning
*S*	Set of sensor nodes
*K*	Number of clusters
*H*	Set of CHs
*M*	Set of CMs
*T*	Minimum percentage of the overlapped region for FoV (Threshold)
*θ*	Vertex angle created by an isosceles triangle
*C_s_*	Congruent side of an isosceles triangle
*λ*	Orientation angle
*ADV*	Advertisement message
*P*	Set of players
*U*	Utility function
*NH*	Set of nodes having higher residual energy
*NL*	Set of nodes having lower residual energy
*E_T_*	Energy threshold
*T_CH_*	Strategy for being a CH
*T_NCH_*	Strategy for not being a CH
*R_i_*	Reward for any node *i*
*R_i_*	Penalty for any node *i*
*α*	The reward to be given to a node in set *NH* if they become CHs
*β*	The reward to be given to a node in set *NL* if they become CHs
*δ*	The step of encouragement or discouragement for being a CH
*ρ*	The rate of nodes in *NH* set to select $T_{CH}$ strategy
*η*	Rate of nodes in *NL* set to select $T_{CH}$ strategy
*N_RE_*	Residual energy level of a node
*I_n_*	Number of rounds
*Q*	Highest number of nodes in a cluster
*I_f_*	Final round
*m*	Any random integer
$RE_{i}$	Residual energy of any node *i*
$Tx_{avg}$	Average number of packets transmitted
$DT_{CH_{i}}$	Data packets transmitted by any CH in the network
$PDR_{avg}$	Average packet delivery ratio
$Rx_{sink}$	Data received by the sink node
*p*	Number of nodes who have higher residual energy
*q*	Number of nodes who have lesser residual energy
*Y*	Number of sink nodes

**Table 2:** Detailed description if the operations in setup and steady state phase.

Phase	Operation
Setup	(1) Cluster formation.
	(2) Intelligent selection of CH nodes using game theory.
	(3) CHs advertise to CM.
	(4) CMs send join requests to CHs.
Steady state	(5) Sink nodes broadcast to CHs.
	(6) CHs decide which sink to transmit data based on the RSSI value.
	(7) Use of data redundancy avoidance algorithm.
	(8) Data transmission.

**Table 3:** Simulation parameters.

Parameter	Value
Network area	100 × 100 m
Number of sensor nodes	100
Data rate	2 Mbps
Image size	176 × 144
Frame rate	30 fps
Offset angle	60°
Sensing radius	30 m
Initial energy of each node	2 J

**Table 4:** Basic energy expenditure of sensor nodes.

Operation	Value
Camera initialization	1725.4 mj
Frame grabbing	537.2 mj
Object recognition	144.2 mj
Shutdown	768.5 mj

**Table 5:** Workload parameters.

Parameter	Value
Traffic load	48 packets/min
Packet size	176 bits (22 bytes)
Transmission rate	2 Mbps
Transmission range	15 m
